# Label-Free Optical Detection of Mycotoxins Using Specific Aptamers Immobilized on Gold Nanostructures

**DOI:** 10.3390/toxins10070291

**Published:** 2018-07-16

**Authors:** Ali Ghanim Al-Rubaye, Alexei Nabok, Gaelle Catanante, Jean-Louis Marty, Eszter Takács, András Székács

**Affiliations:** 1 Materials and Engineering Research Institute, Sheffield Hallam University, Sheffield S1 1WB, UK; a.nabok@shu.ac.uk; 2 Basra Technical Institute, Southern Technical University, 61002 Basra, Iraq; 3 Department of Biochemistry and Molecular Biology, University of Perpignan, 66100 Perpignan, France; gaelle.catanante@univ-perp.fr (G.C.); jlmarty@univ-perp.fr (J.-L.M.); 4 Agro-Environmental Research Institute, NARIC, 1011-1239 Budapest, Hungary; e.takacs@cfri.hu (E.T.); a.szekacs@cfri.hu (A.S.)

**Keywords:** optical label-free biosensor, mycotoxins, aptamer, LSPR, gold nanostructures, total internal reflection ellipsometery

## Abstract

This work focuses on the development of the novel label-free optical apta-sensors for detection of mycotoxins. A highly sensitive analytical method of total internal reflection ellipsometry (TIRE) combined with Localized Surface Plasmon Resonance (LSPR) phenomenon in nano-structured gold films was exploited here for the first time for detection of aflatoxin B1 and M1 in direct assay with specific aptamers immobilized on the surface of gold. The achieved detection of low molecular weight molecules, such as aflatoxin B1 and M1, in a wide range of concentrations from 100 ng/mL down to 0.01 ng/mL is remarkable for the LSPR method. The study of binding kinetics of aflatoxin molecules to their respective aptamers using dynamic TIRE measurements yielded the values of affinity constants in the range of 10^−8^–10^−7^ mol, which is characteristic for highly specific aptamer/target interactions similar to that for monoclonal antibodies. The effect of aptamers’ DNA chain length on their binding characteristics was analyzed.

## 1. Introduction

This work is dedicated to the detection of mycotoxins produced by different fungi species that may grow on various agricultural products, such as grains, nuts, coffee beans, spices, fruits, and associated food and animal feed, stored at inappropriate conditions, i.e., elevated temperatures and high humidity [1]. Because of the toxic, carcinogenic, and endocrine disruptive properties of mycotoxins health and environmental control organizations worldwide established very low limits (typically in sub-ppb level of concentrations) for mycotoxins in food and feed, which makes their detection extremely difficult, especially considering their low molecular weight [2]. Although the modern analytical methods such as HPLS or mass spectroscopy can cope with these demands, the above mentioned methods are expensive and time consuming. Therefore the development of biosensors suitable for express and cost-effective detection of mycotoxins is of great demand nowadays [3]. Optical biosensors lead this development with a number of biosensors based on the different instrumentation and principles (i.e., Surface Plasmon Resonance (SPR), Total Internal Reflection Ellipsometry (TIRE), Optical Waveguide Lightmode Spectroscopy (OWLS), Mach–Zehnder (MZ) interferometers, etc.) [3]. We would like to highlight the method of total internal reflection ellipsometry (TIRE) which was developed in the last 10–15 years, as a highly sensitive analytical tool in bio-sensing, particularly attractive for the detection of low molecular weight analytes, and mycotoxins in particular [4,5,6,7,8]. Another interesting optical phenomenon of localized surface plasmon resonance (LSPR) [9] has recently attracted the attention of researches because of its potentially high sensitivity allowing the detection of single molecules [10]. Localized surface plasmon resonance (LSPR) is the phenomenon related to the collective oscillation of conducting electrons in resonance with incident an electromagnetic field that typically appear in metal nanostructures with dimensions comparable to the wavelength of light. LSPR is typically manifested as an absorption band in the middle of visible spectral range [11], position of which is affected by refractive index of the medium; the latter fact actually constitutes the principle of LSPR sensing since small changes of local refractive index due to absorption of molecules cause a shift of LSPR band to higher wavelengths [12]. The sensitivity of LSPR sensors based on UV-vis absorption spectroscopy is not very high and limit the use of LSPR for the detection of rather large protein molecules. A combination of LSPR and TIRE methods increases the sensitivity further and allows the detection of small molecules such as mycotoxins. The detection of aflatoxin B1 in concentrations down to 0.1 ng/mL was achieved recently in immunoassay with specific monoclonal antibodies using a combination of LSPR and TIRE [13].

One of the obstacles to achieving a high sensitivity of LSPR via immunosensors lies in much smaller evanescent field decay length as compared to traditional SPR [14,15]. From that point of view, the use of artificial bio-receptors, such as aptamers having much smaller dimensions than antibodies could be beneficial. Aptamers are short single strand DNA or RNA oligomers synthesized to accommodate particular target molecules of inorganic, organic, and bio-organic origin by selecting nucleotides from a large random sequence pool using SELEX process [16,17]. Generally, aptamers are getting increasingly common in biosensing, effectively replacing antibodies [18]. Aptamers can be immobilised on the surface via functional groups, such as thiols, on one end and may contain either fluorescent or redox labels on the other end, and are therefore used in optical or electro-chemical biosensors.

We recently reported on the use of aptamers in conjunction with the TIRE method for the detection of ochratoxin A. Where the achieved low detection limit (LDL) was in the range of 0.01 ng/mL [19]. The novel and important part of that work was the use of unlabeled aptamers. One of the advantages of our sensing technology, e.g., ellipsometry, is in the high accuracy of measurements of the thin films’ thickness. Where the process of aptamers wrapping round the target molecules is accompanied by reduction in the aptamer layer thickness, which was actually recorded in our experiments [19]. In this work, we attempted the detection of aflatoxins B1 and M1 in assay with specific aptamers using a combination of the Localized Surface Plasmon Resonances phenomenon in nanostructured gold films with the method of TIRE.

## 2. Results and Discussion

### 2.1. Study of Aflatoxin/Aptamer Binding Using LSPR/TIRE

**Figure 1:**
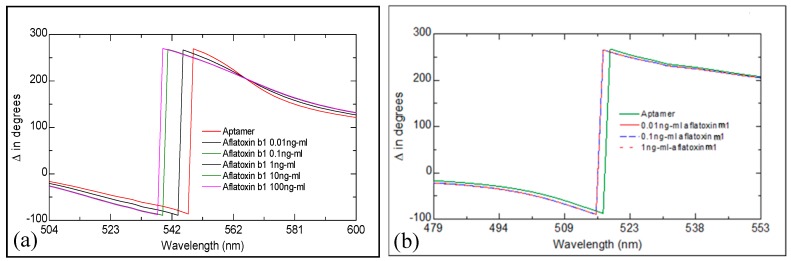
Series of TIRE ∆ spectra recorded upon biding AFT B1 (**a**) and AFT M1 (**b**) to respective specific aptamers.

Figure 1a shows a set of Δ spectra recorded on our LSPR transducers functionalized with anti- aftatoxin (anti-AFT) B1 after consecutive binding steps of aflatoxin B1 starting from small concentrations. A progressive “blue” (to shorter wavelength) spectral shift was observed, which is typically associated with the reduction in the aptamer layer thickness due to aptamers coiling around the target molecules. Similar results were obtained for binding aflatoxin M1 to its specific aptamer (see Figure 1b), however the saturation of the response was observed at the much smaller concentrations of AFT M1 of 1 ng/mL, which could be caused by a smaller concentration of active aptamers immobilized on the surface. In addition, the cross-sensitivity of the two aptamers was tested, and no spectral shift was recorded when attempted binding AFT B1 to an aptamer specific to AFT M1, and vice versa. Also, both the anti-AFT B1and anti-AFT M1 aptamers do not bind OTA (ochratoxin A).

**Figure 2:**
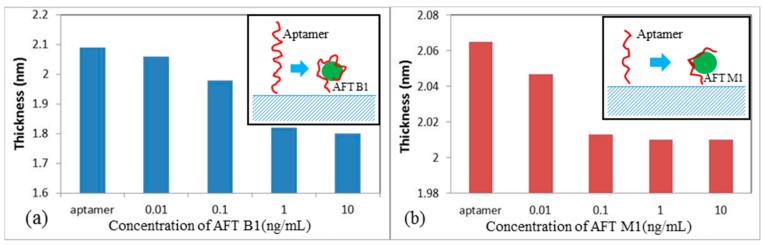
Dependences of the aptamer layer thickness vs concentration of AFT B1 (**a**) and AFT M1 (**b**). The processes of aptamer/target binding are illustrated by the corresponding insets.

The fitting of the TIRE spectra to a three layer model [13], allowed the evaluation of the aptamer layer thickness that is shown in Figure 2. The absolute values of the aptamer layer thickness, which depends on several factors, i.e., aptamer chain length, concentration of aptamers, concentration of MgCl_2_ in the buffer, etc., are not informative actually. The main interest was in the changes of aptamer layer thickness upon binding the target molecules. The decrease in the anti-AFT B1 aptamer layer thickness (14.2%), upon increasing the concentration of AFT B1, is much more noticeable (Figure 2a) than that (2.6%) for the layer of anti-AFT M1 binding the respective target (Figure 2b).

As was suggested, the decrease in the aptamer layer thickness upon binding aflatoxins is caused by coiling aptamers around the target molecules, and this process is schematically illustrated on inset in Figure 2a. The differences in the binding abilities of anti-AFT B1 and anti-AFT M1 are most-likely related to their oligonucleotides chain length (50 and 21, respectively). The much shorter aptamer to AFT M1 may not fully wrap the target molecule, but rather have a small bend in the chain (see inset in Figure 2b), which is still detectable using a sensitive ellipsometry instrument. The spectral resolution of our ellipsometry instrument did not allow the detection of aflatoxins below 0.01 ng/mL. Nevertheless, the minimal detected concentration of 0.01 ng/mL for both aflatoxins corresponded to part per billion, which is a remarkable result for direct aptamer assay format.

### 2.2. Study of Aflatoxin-Aptamer Binding Kinetics

**Figure 3:**
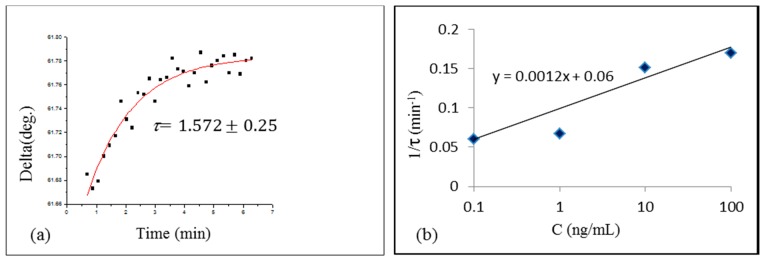
Typical time dependence of ∆ during binding AFT B1 of 1 ng/mL to aptamers (**a**); evaluation of the association constant *K_A_* (**b**).

Following the procedure described earlier [6,7,8,13], the study of the kinetics of binding AFT B1 to a specific aptamer was carried out by recording a number of TIRE spectra during binding reaction, and then plotting the time dependences of the values of Ψ or Δ at the particular wavelength. Figure 3a shows a typical time dependence of Δ at 600 nm during binding of 10 ng/mL of AFT B1 to specific aptamer. The time constant *τ* was evaluated by fitting the data to the rising exponential function. The same procedure was repeated for different concentrations of AFT B1, and the obtained values of *τ* were plotted as 1/*τ* against AFT B1 concentration (C) in Figure 3b.

The absorption rate ($k_{a}$) and desorption rate ($k_{d}$) were found, respectively, from the gradient and intercept of the linear graph 1/*τ* = $k_{a}\;C+k_{d}\;$in Figure 3b. Then, the association and affinity constants were found as *K_A_* = *k_a_*/*k_d_* and *K_D_ =* 1/*K_A_*. The obtained values of *K_A_ =* 3.12·10^7^ (mol^−1^) and *K_D_ =* 3.2 × 10^−8^ (mol) shows high specificity of AFT B1/aptamer interaction, similar to that of monoclonal antibodies to AFT B1 [8].

Asimilar analysis was carried out for binding the kinetics of AFT M1 to its respective aptamer and yielded the values of *K_A_* = 4.64 × 10^6^ (mol^−1^) and *K_D_* = 2.1 × 10^−7^ (mol). The obtained values of the affinity coefficient for anti-AFT B1 are similar to those reported in [17,20]. The affinity of a shorter anti-AFT M1 aptamer appeared to be much smaller than that of anti-AFT B1 having a longer chain. This proved our initial suggestion of the effect of the aptamer nucleotide chain length on its affinity.

## 3. Conclusions

The combination of a simple LSPR transducer based on nanostructured gold films and the optical method of total internal reflection ellipsometry (TIRE) allowed for label-free detection of small molecules of aflatoxin in direct assay with specific aptamers immobilized on the surface of gold. The observed decrease in the aptamer layer thickness upon the binding of their specific target molecules directly confirms the quite obvious model of aptamers wrapping round the target. The concentration range for aflatoxin B1 detection stretches from 0.01 ng/mL to 100 ng/mL, where the minimal detected concentration of aflatoxins corresponding to 0.01 ppb is remarkably low for LSPR-based biosensors, especially considering the small molecular weight of aflatoxin toxin molecules and direct aptamer assay format. Such sensitivity, although it may not be as good as that for advance methods of HPLC or mass spectroscopy, is suitable for the detection of mycotoxins in concentrations close to the legislated limits [2], but the cost and time of the analysis is definitely much lower. The study of binding kinetics of AFT B1 and AFT B1 to specific aptamers confirmed the high specificity of the aptamer/target reaction with the affinity constants found to be in tens of nano-moles, which are similar to the values reported earlier [17,20] and similar to those of antigen/antibody reactions [5,6,7,8]. The shorter-chain aptamer to AFT M1 appeared to be less specific as compared to longer-chain aptamer to AFT B1. To our knowledge, this is the first report on the experimental evaluation of aptamers affinity.

## 4. Experimental Details

### 4.1. TIRE Experimental Set-Up

**Figure 4:**
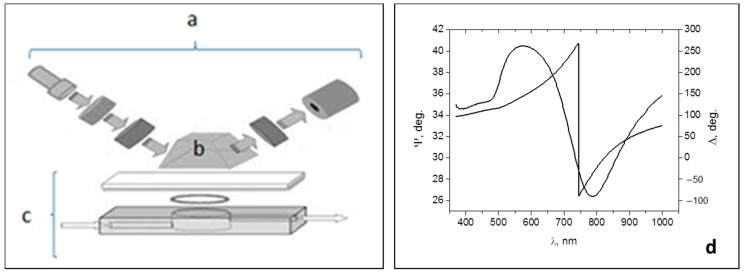
Schematic diagram of TIRE experimental set-up: spectroscopic ellipsometer M2000 (**a**), 68° prism (**b**), reaction cell (**c**). Typical TIRE spectra of 25 nm thick Au film deposited on glass (**d**). Reproduced from [5], Copyright 2008, Elsevier, New York, NY, USA.

The TIRE experimental set-up shown schematically in Figure 4a–c is based on M2000, J.A. Woollam, spectroscopic ellipsometer (a) operating in the 370–1680 nm wavelength range. The principles of TIRE were described in detail previously [4,5,6,7,8]. Briefly, the method of TIRE combines the advanced spectroscopic ellipsometry instrumentation with traditional Kretschmann SPR geometry. The addition of a 68° glass prism (b) provides the conditions of total internal reflection for the coupling of light into thin gold film (either continuous or nano-structured) deposited on glass. A 0.2 mL PTFE cell (c) is attached to the sample and allows the injection of different solutions into the cell to study different biochemical reactions. The TIRE method has an advantage of recoding two ellipsometric parameters Ψ and Δ, which represent, respectively, the amplitude ratio and phase shift between *p*- and *s*-components of polarized light.

Figure 4d shows typical TIRE spectra of Ψ and Δ recorded on continuous 25 nm thick gold film deposited on glass, where Ψ spectrum resembles a traditional SPR curve, while Δ spectrum represents a phase drop near the resonance [5]. The parameter of Δ is particularly sensitive to changes in the refractive index of a medium, and thus, to molecular adsorption. Small changes in either thickness or refractive index of the molecular layer deposited on gold cause the shift of the Δ spectrum; the values of thickness can be obtained by the data fitting and can be subsequently used as the TIRE sensor output [5]. In addition to standard TIRE spectroscopic measurements, we can record the time dependencies of Ψ and Δ at particular wavelengths to study the kinetics of molecular adsorption or binding [5,6,7,8].

### 4.2. Preparation of LSPR Transducers

**Figure 5:**
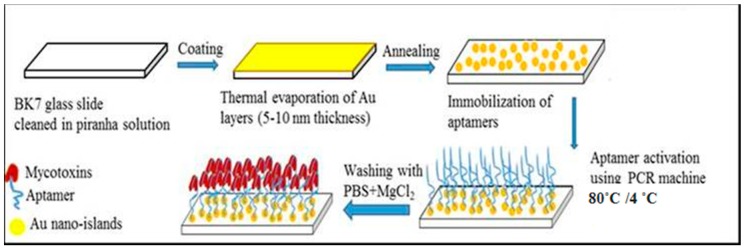
The sequence of steps of samples preparation.

The fabrication of nanostructured gold films for LSPR experiments is schematically outlined in Figure 5. Standard microscopic glass slides are cleaned with hot “piranha” solution (1:3 H_2_O_2_/H_2_SO_4_) for 1 h, then rinsed with deionized water and dried under a stream of nitrogen gas. A layer of gold was thermally evaporated on clean glass slides in 10^−7^ Torr vacuum using Edwards 360 metal evaporation unit. The thickness of Au layers was in the range from 5 nm to 10 nm and controlled by Quartz Crystal Microbalance QCM sensor during evaporation. The gold coated slides were then annealed in air at 550–580 °C for 10 h as described in [10], which transforms the continuous gold films into nanostructures consisting of randomly distributed gold islands with the average lateral dimensions of 100 to 200 nm and exhibiting a distinctive LSPR band in visible spectral range [10,13,15].

### 4.3. Aptamers and other Chemicals

The aptamers used in our work were synthesized and purified by Microsynth (Schutzenstrasse, Balgach, Switzerland). The sequence of nucleotides for anti-aflatoxin B1 (AFT B1) and anti-aflatoxin M1 (AFT M1) aptamers, which were established previously in [17,20] are shown below:

5′SH-GTTGGGCACGTGTTGTCTCTCTGTGTCTCGTGCCCTTCGCTAGGCCCACA-3′ for AFT B1, and 5′SH-ACTGCTAGAGATTTTCCACAT-3′ for AFT M1.

For our purposes, we use aptamers with no labels on 3′ termini, but only SH groups at 5′ termini for immobilization on the surface of gold.

Aflatoxin B1 and M1 were purchased from Sigma-Aldrich. The other chemicals used were magnesium chloride (MgCl_2_), Dithiothreitol (DTT) from Sigma-Aldrich. All reagents were of analytical grade. Deionized Milli-Q water was used for preparation of reagents throughout the experiments.

Hepes binding buffer (HBB, 50 mM) was prepared by dissolving 50 mM Hepes sodium salt (3 mM MgCl_2_, 120 mM NaCl, and 5 mM KCl) in deionized Milli-Q water. The pH of the buffer was adjusted to 7.4. Similarly, phosphate binding buffer (PBB, 100 mM) was prepared by dissolving 10 mM Na_2_HPO_4_, 1.76 mM KH_2_PO_4_, 3 mM MgCl_2_, 2.7 mM KCl, and 137 mM NaCl in deionized water. The pH of the buffer was adjusted to 7.4. The presence of MgCl_2_ in both HBB and PBB is crucial for preventing self-coiling of aptamers.

The aptamers are delivered in lyophilized form. Therefore, aptamer stock solution is prepared at 100 µM by adding an appropriate volume of de-ionized water. For long-term storage, as-received aptamers were prepared in small 100 µM aliquots in deionized water and stored frozen. The stock solution of aptamer was diluted at the desired concentration with HBB or PBB supplemented with 1 mM of DTT and then subjected to heating 90 °C for 5 min and cooling 4 °C for 5 min cycle in PCR machine before use.

Aflatoxin B1 and M1 stock solution (1 mg·mL^−1^) were prepared in acetonitrile. In addition, further diluted solutions were prepared in HBB or PBS. We maintained the percentage of acetonitrile at less than 2% in the final assay throughout the experimental procedure. All of the working solutions were prepared freshly before use and stored at 4 °C when not in use.

### 4.4. Immobilization of Aptamers on Gold

Immobilization of anti-AFT B1 and anti-AFT M1 aptamers on nanostructured gold films was carried out following the protocols described in detail in [18,19,21]. As shown in Figure 5, a 100 µL of respective aptamer solutions of optimized concentration 5 µM or 2 µM in PBB or HBB buffer supplemented with 1 mM of DTT was pipetted onto prepared glass slides with nano-structured gold. After incubation for 4 h in a humidity chamber, the slides were rinsed with PBB to remove unbound aptamers. The resultant aptamer modified slides were either used directly as aptasensor or stored in binding buffer at 4 °C for several days without any noticeable decrease in their functionality.
